# Supplementary material for: The Evolution and Characterization of the RNA Interference Pathways in Lophotrochozoa
Source: Genome Biol Evol. 2024 May 7;16(5):evae098. doi: 10.1093/gbe/evae098 (PMC11114477; doi:10.1093/gbe/evae098)
Supplement: evae098_Supplementary_Data [file evae098_supplementary_data.zip › Supplementary_materials.zip!/selected_miRNAs/NoGe_selected_miRNAs/mirdeep_pdf_files/NMRB01000380.1_Notospermus_geniculatus_strain_Ushimado_scaffold380,_whole_genome_shotgun_sequence_11924.pdf]

Provisional ID	:	NMRB01000380.1_Notospermus_geniculatus_strain_Ushimado_scaffold380,_whole_genome_shotgun_sequence_11924
Score total	:	42727.8
Score for star read(s)	:	3.9
Score for read counts	:	42718.9
Score for mfe	:	2
Score for randfold	:	0
Score for cons. seed	:	3
Total read count	:	83803
Mature read count	:	83505
Loop read count	:	0
Star read count	:	298

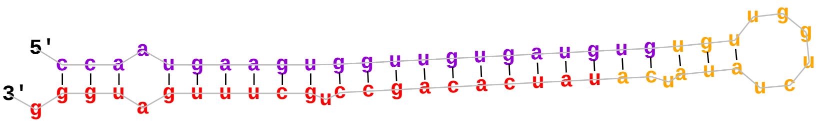

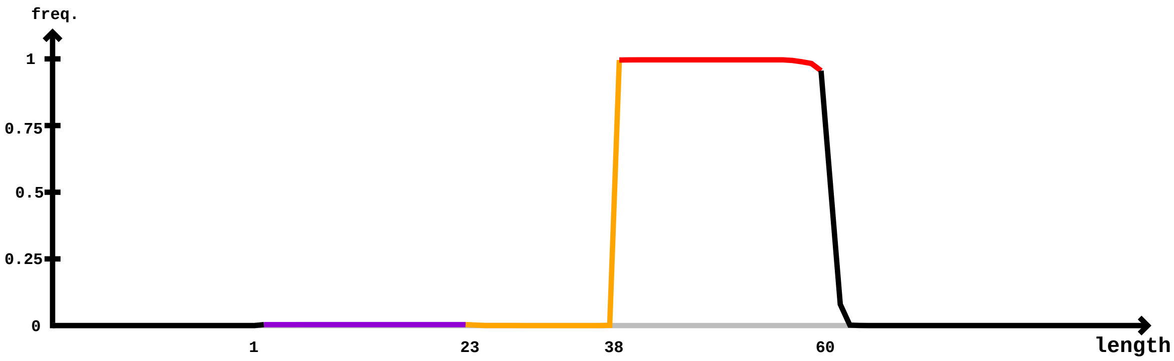

Star **Mature**

```
5'- augcauacaguuuguauaguccaaugaagugguugugauguguguuggucuauaucauaucacagccugcuuugaugggcuauacugcuguuuucugccaagaaggguucuu   -3'   obs
    augcauacaguuuguauaguccaaugaagugguugugauguguguuggucuauaucauaucacagccugcuuugaugggcuauacugcuguuuucugccaagaaggguucuu         exp
    ......(((((..((((((((((.(((((((((((((((((((((......))).)))))))))))).)))))).)))))))))).)))))(((((....))))).......   reads   mm   sample
    ....................ccaaugaagugguuguAaugug......................................................................   1       1    Fm2
    ....................ccaaugaagugguugugaugug......................................................................   47      0    Fm2
    ....................ccaaugUagugguugugaugug......................................................................   1       1    Fm2
    ....................ccaaugaagugguugugauguguguCg.................................................................   1       1    Fm2
    ....................Ucaaugaagugguugugauguguguug.................................................................   1       1    Fm2
    ....................ccaaugaagugguugugauguguguug.................................................................   20      0    Fm2
    ........................................................Cuaucacagccugcuuugauggg.................................   8       1    Fm2
    ........................................................auaucacagccugcuuugauggg.................................   9       0    Fm2
    .........................................................uaucacagccugcuuuga.....................................   5       0    Fm2
    .........................................................uaucacagccugcuuugUu....................................   4       1    Fm2
    .........................................................uaucacagccugcuuugau....................................   36      0    Fm2
    .........................................................uaucacagccugUuuugaug...................................   8       1    Fm2
    .........................................................uaucacagccugcuuugauU...................................   2       1    Fm2
    .........................................................uaucacagccugcuuugaug...................................   59      0    Fm2
    .........................................................uaucacagccugcuuGgaug...................................   1       1    Fm2
    .........................................................uaucacagccugcuuugUug...................................   1       1    Fm2
    .........................................................uaucacagccugcuuugGugg..................................   1       1    Fm2
    .........................................................uaucacagccugcuuugCugg..................................   3       1    Fm2
    .........................................................uaucacagccugcuuugaugg..................................   199     0    Fm2
    .........................................................uaucacagccugcuGugauggg.................................   1       1    Fm2
    .........................................................uauAacagccugcuuugauggg.................................   2       1    Fm2
    .........................................................uUucacagccugcuuugauggg.................................   2       1    Fm2
    .........................................................uaucaNagccugcuuugauggg.................................   3       1    Fm2
    .........................................................uaucacagccAgcuuugauggg.................................   21      1    Fm2
    .........................................................uaucacagccGgcuuugauggg.................................   11      1    Fm2
    .........................................................Caucacagccugcuuugauggg.................................   1       1    Fm2
    .........................................................uaucacGgccugcuuugauggg.................................   11      1    Fm2
    .........................................................Naucacagccugcuuugauggg.................................   4       1    Fm2
    .........................................................uaucaUagccugcuuugauggg.................................   3       1    Fm2
    .........................................................uaucacagccugcuuugauggU.................................   19      1    Fm2
    .........................................................uaucacagccugUuuugauggg.................................   1       1    Fm2
    .........................................................uaucacaUccugcuuugauggg.................................   1       1    Fm2
    .........................................................uaucacCgccugcuuugauggg.................................   2       1    Fm2
```

```
                   Star                                Mature
augcauacaguuuguauaguccaaugaagugguugugauguguguuggucuauaucauaucacagccugcuuugaugggcuauacugcuguuuucugccaagaaggguucuu
........................................................uaucacagccugcCuugauggg..................................    3     1    Fm2
........................................................uGucacagccugcuuugauggg..................................    1     1    Fm2
........................................................uaucCcagccugcuuugauggg..................................    2     1    Fm2
........................................................uaucacNgccugcuuugauggg..................................    1     1    Fm2
........................................................uaucacagccugcuuugauUgg..................................    2     1    Fm2
........................................................uauGacagccugcuuugauggg..................................    1     1    Fm2
........................................................uaucacagccugcuuuAauggg..................................    16    1    Fm2
........................................................uaucacagccugcuuugaugUg..................................    4     1    Fm2
........................................................uaucacaCccugcuuugauggg..................................    1     1    Fm2
........................................................uaucacagccugcuuugauCgg..................................    1     1    Fm2
........................................................uaucacagccugcuuuUauggg..................................    12    1    Fm2
........................................................uaucacagccugcuuugGuggg..................................    24    1    Fm2
........................................................uaucacagccugcuuugUuggg..................................    19    1    Fm2
........................................................uaucacagccuAcuuugauggg..................................    4     1    Fm2
........................................................uaucacagccugcuuugaAggg..................................    2     1    Fm2
........................................................uaucacagccugcuuuCauggg..................................    37    1    Fm2
........................................................uaucacagccugcuuugCuggg..................................    50    1    Fm2
........................................................uaucacagcUugcuuugauggg..................................    18    1    Fm2
........................................................uaucacagccugcAuugauggg..................................    2     1    Fm2
........................................................uaucacagcAugcuuugauggg..................................    1     1    Fm2
........................................................uaucacagccugcuuugaugCg..................................    1     1    Fm2
........................................................uaucacagccugcGuugauggg..................................    2     1    Fm2
........................................................uaucacagccugcuCugauggg..................................    2     1    Fm2
........................................................uaCcacagccugcuuugauggg..................................    2     1    Fm2
........................................................uaucacagccugcuuugauggA..................................    35    1    Fm2
........................................................uaGcacagccugcuuugauggg..................................    2     1    Fm2
........................................................uaucacagccugcuuugauAgg..................................    1     1    Fm2
........................................................uaucacagccugcuuugaCggg..................................    1     1    Fm2
........................................................uaucacagUcugcuuugauggg..................................    1     1    Fm2
........................................................uaucacagccCgcuuugauggg..................................    4     1    Fm2
........................................................uaucacagccugcuuugauggC..................................    4     1    Fm2
........................................................uaAcacagccugcuuugauggg..................................    3     1    Fm2
........................................................uaucGcagccugcuuugauggg..................................    2     1    Fm2
........................................................uaucacagccugcuuugauggg..................................    8013  0    Fm2
........................................................uaucacagccugcuuugaugggA.................................    1903  1    Fm2
........................................................uaucacagccugcuuugaugggU.................................    223   1    Fm2
........................................................uaucacagccugcuuugauggUc.................................    2     1    Fm2
........................................................uauGacagccugcuuugaugggc.................................    1     1    Fm2
........................................................uaucacagccugcuuuUaugggc.................................    1     1    Fm2
........................................................uaucacagccugcuuugUugggc.................................    2     1    Fm2
........................................................uaucacagccGgcuuugaugggc.................................    1     1    Fm2
........................................................uaucacagccugcuuugCugggc.................................    2     1    Fm2
........................................................uaucacagccugcuuugaugCgc.................................    2     1    Fm2
........................................................uaucacaCccugcuuugaugggc.................................    1     1    Fm2
........................................................uaucacagccugcuuuCaugggc.................................    2     1    Fm2
........................................................uaucacagccugcuuugaugggc.................................    559   0    Fm2
........................................................uaucacagcUugcuuugaugggc.................................    11    1    Fm2
........................................................uaucacagccAgcuuugaugggc.................................    3     1    Fm2
........................................................uaucacagccugcuuugaugggG.................................    81    1    Fm2
........................................................uaucacagccugcuuugaugUgc.................................    10    1    Fm2
........................................................uaucacagUcugcuuugaugggc.................................    26    1    Fm2
........................................................uaucacagccugcuuugGugggcu................................    1     1    Fm2
........................................................uaucacagUcugcuuugaugggcu................................    10    1    Fm2
........................................................uaucacagccugcuuugaugggAu................................    66    1    Fm2
........................................................uaucacagccugcuuugaugggcG................................    7     1    Fm2
........................................................uaucacagccugcuuugaugggUu................................    15    1    Fm2
........................................................uaucacagccugcuuugaugggcu................................    81    0    Fm2
........................................................uaucacagccugcuuugaugggcua...............................    11    0    Fm2
.........................................................aucacagccugcuuugauggg..................................    1     0    Fm2
..........................................................ucacagccAgcuuugaugg...................................    1     1    Fm2

....................ccaaugaagugguugugaugug......................................................................    35    0    Fm1
........................................................uaucacagccugcuuuga......................................    5     0    Fm1
........................................................uaucacagccugcuuugau.....................................    54    0    Fm1
........................................................uaucacagUcugcuuugau.....................................    2     1    Fm1
........................................................uaucacagccugcuuugaug....................................    61    0    Fm1
........................................................uaucacagccugcuuugauU....................................    7     1    Fm1
........................................................uaucacagccugcuuugauC....................................    1     1    Fm1
........................................................uaucacagUcugcuuugaug....................................    4     1    Fm1
........................................................uaucacagccugcuuugauA....................................    5     1    Fm1
```

```
                    Star                                   Mature
augcauacaguuuguauaguccaaugaaguggguugugauguguguuggucuauaucauaucacagccugcuuugaugggcuauacugcuguuuucugccaagaaggguucuu
.........................................................Caucacagccugcuuugaug...................................  1     1  Fm1
.........................................................uaucacagccAgcuuugaugg..................................  9     1  Fm1
.........................................................uaucacagccugcuuugaugU..................................  1     1  Fm1
.........................................................uaucacagccugcuuCgaugg..................................  1     1  Fm1
.........................................................uauAacagccugcuuugaugg..................................  1     1  Fm1
.........................................................uaucacagUcugcuuugaugg..................................  1     1  Fm1
.........................................................uaucacNgccugcuuugaugg..................................  1     1  Fm1
.........................................................uaucacagccugcuuugaugg..................................  246   0  Fm1
.........................................................uaucacagccugcuuugUugg..................................  1     1  Fm1
.........................................................uaucacagccugcuuugauUg..................................  1     1  Fm1
.........................................................uaucacagcGugcuuugauggg.................................  1     1  Fm1
.........................................................uUucacagccugcuuugauggg.................................  1     1  Fm1
.........................................................uaucCcagccugcuuugauggg.................................  2     1  Fm1
.........................................................uaucacagccugcuuugaCggg.................................  1     1  Fm1
.........................................................uaucacagccugcuuugaugUg.................................  3     1  Fm1
.........................................................uaucacagccugcuuuUauggg.................................  5     1  Fm1
.........................................................uaucacagccugcAuugauggg.................................  1     1  Fm1
.........................................................uaucacaAccugcuuugauggg.................................  1     1  Fm1
.........................................................uaucacagccCgcuuugauggg.................................  1     1  Fm1
.........................................................uaucacagccugcuuuCauggg.................................  15    1  Fm1
.........................................................uaucacagcUugcuuugauggg.................................  10    1  Fm1
.........................................................uaucacagccugcuuugauggA.................................  17    1  Fm1
.........................................................uaucacagccugcuuugauggU.................................  15    1  Fm1
.........................................................uaucacagccugcuuugGuggg.................................  9     1  Fm1
.........................................................uaucGcagccugcuuugauggg.................................  1     1  Fm1
.........................................................uaucacagccugcuuugCuggg.................................  18    1  Fm1
.........................................................uaucacUgccugcuuugauggg.................................  1     1  Fm1
.........................................................uaucacagccGgcuuugauggg.................................  3     1  Fm1
.........................................................uauGacagccugcuuugauggg.................................  1     1  Fm1
.........................................................uaucacagccugcuuuAauggg.................................  3     1  Fm1
.........................................................uaucacagccAgcuuugauggg.................................  5     1  Fm1
.........................................................Naucacagccugcuuugauggg.................................  2     1  Fm1
.........................................................uaucacagccugcuuugauggg.................................  3221  0  Fm1
.........................................................uaucacagccugcuuugauggC.................................  25    1  Fm1
.........................................................uaucacagccuCcuuugauggg.................................  1     1  Fm1
.........................................................uaucacagccugcuuugauUgg.................................  1     1  Fm1
.........................................................uaucacagccugcuuugUuggg.................................  10    1  Fm1
.........................................................uauUacagccugcuuugauggg.................................  2     1  Fm1
.........................................................uaucacagcUugcuuugaugggc................................  25    1  Fm1
.........................................................uaucacagccugcuuugUugggc................................  8     1  Fm1
.........................................................uUucacagccugcuuugaugggc................................  1     1  Fm1
.........................................................uaucaUagccugcuuugaugggc................................  1     1  Fm1
.........................................................uaucacagccugUuuugaugggc................................  5     1  Fm1
.........................................................Naucacagccugcuuugaugggc................................  1     1  Fm1
.........................................................uaucacagccugcuuuAaugggc................................  8     1  Fm1
.........................................................uaucacagccuUcuuugaugggc................................  1     1  Fm1
.........................................................uaucacagccugcuuuUaugggc................................  5     1  Fm1
.........................................................uaucacaAccugcuuugaugggc................................  1     1  Fm1
.........................................................uaucacagccugcuuugaugCgc................................  1     1  Fm1
.........................................................uaucacagccugcuuugaugUgc................................  10    1  Fm1
.........................................................uaucacagccugcuuugaugggU................................  219   1  Fm1
.........................................................uaucacagccugcuuugaugggc................................  3732  0  Fm1
.........................................................uaucacagccugcCuugaugggc................................  2     1  Fm1
.........................................................uaucacagccGgcuuugaugggc................................  3     1  Fm1
.........................................................uaucacGgccugcuuugaugggc................................  4     1  Fm1
.........................................................uaucacagccugcuuugCugggc................................  11    1  Fm1
.........................................................uaucacagccCgcuuugaugggc................................  3     1  Fm1
.........................................................uaucacagccugcuuugauggAc................................  2     1  Fm1
.........................................................uaucacagccugcuuugaugggA................................  548   1  Fm1
.........................................................uaucacaUccugcuuugaugggc................................  1     1  Fm1
.........................................................uaucacagccugcuuugauggCc................................  1     1  Fm1
.........................................................uaucacagccugcuuugaAgggc................................  1     1  Fm1
.........................................................uaucacagccugcuuugaugggG................................  24    1  Fm1
.........................................................uaucacagUcugcuuugaugggc................................  23    1  Fm1
.........................................................uaucacagccugcuuugGugggc................................  10    1  Fm1
.........................................................uaucacagccAgcuuugaugggc................................  9     1  Fm1
.........................................................uaucacagccugcuuuCaugggc................................  14    1  Fm1
.........................................................uaucacagccugcuuugauUggc................................  2     1  Fm1
.........................................................uaucacagccugcuCugaugggc................................  1     1  Fm1
.........................................................uaucacagccugcuuugaCgggc................................  1     1  Fm1
```

Star **Mature**

augcauacaguuuguauaguccaaugaagugguugugauguguguuggucuauaucauaucacagccugcuuugaugggcuauacugcuguuuucugccaagaaggguucuu			
....uaucacagUcugcuuugaugggcu...............................	4	1	Fm1
....uaucacagccugcuuugCugggcu...............................	1	1	Fm1
....uaucacagccugcuuugauUggcu...............................	1	1	Fm1
....uaucacagccAgcuuugaugggcu...............................	1	1	Fm1
....uaucacagccugcuuugaugggcA...............................	8	1	Fm1
....uaucaUagccugcuuugaugggcu...............................	1	1	Fm1
....uaucacagccugcuuugaugggAu...............................	6	1	Fm1
....uaucacagccGgcuuugaugggcu...............................	1	1	Fm1
....uaucacagccugUuuugaugggcu...............................	1	1	Fm1
....uaucacagcUugcuuugaugggcu...............................	3	1	Fm1
....uaucacagccugcuuugaugggcC...............................	1	1	Fm1
....uaucacagccugcuuugaugUgcu...............................	1	1	Fm1
....uaucacagccugcuuuAaugggcu...............................	1	1	Fm1
....uaucacagccugcuuuUaugggcu...............................	3	1	Fm1
....uaucacagccugcuuugaugggcG...............................	48	1	Fm1
....uaucacagccugcuuuCaugggcu...............................	3	1	Fm1
....uaucacagccugcuuugauggAcu...............................	1	1	Fm1
....Caucacagccugcuuugaugggcu...............................	1	1	Fm1
....uaucacaAccugcuuugaugggcu...............................	1	1	Fm1
....uaucacagccugcuuugaugggUu...............................	27	1	Fm1
....uaucacagccugcuuugUugggcu...............................	1	1	Fm1
....uaucacagccuUcuuugaugggcu...............................	1	1	Fm1
....uaucacGgccugcuuugaugggcu...............................	2	1	Fm1
....uaucacagccugcuuugaugggcu...............................	781	0	Fm1
....uaucacagccugcuuugGugggcu...............................	2	1	Fm1
....uaucacagccugcuuugaugggcuU..............................	28	1	Fm1
....uaucacagccugcuuugaugggcuau.............................	3	0	Fm1
....uaucacagccugcuuugaugggcuUu.............................	10	1	Fm1
....aucacagccugcuuugau....................................	2	0	Fm1
....ucacagccAgcuuugaugg..................................	1	1	Fm1
....uaucacagccugcuuuga.....................................	36	0	Mm1
....uaucacagccugcuuuCa.....................................	1	1	Mm1
....uaucacagccugcuuugU.....................................	1	1	Mm1
....uaucacagccugcuuAga.....................................	1	1	Mm1
....uaucacagccugcuuugUu....................................	7	1	Mm1
....uaucacagccugcuuugaug...................................	16	0	Mm1
....uaucacagccugcuuGgaug...................................	1	1	Mm1
....uaucacagccugcuuugaugg..................................	122	0	Mm1
....uaucacagccugcuuugGugg..................................	1	1	Mm1
....uaucacagccugcuuugaugU..................................	4	1	Mm1
....uaucacagccAgcuuugauggg.................................	3	1	Mm1
....uaucacagccugcuCugauggg.................................	1	1	Mm1
....uaucacagUcugcuuugauggg.................................	2	1	Mm1
....uaCcacagccugcuuugauggg.................................	1	1	Mm1
....uaucacagccugcuuugauggA.................................	18	1	Mm1
....uaucacagccugcuuuCauggg.................................	6	1	Mm1
....uaucGcagccugcuuugauggg.................................	1	1	Mm1
....uaucacagccCgcuuugauggg.................................	3	1	Mm1
....uaucacagccugcuuugauggg.................................	1282	0	Mm1
....uaucacagccGgcuuugauggg.................................	1	1	Mm1
....uGucacagccugcuuugauggg.................................	2	1	Mm1
....uaucacagccugcuuuAauggg.................................	3	1	Mm1
....uaucacagccugcuuugUuggg.................................	6	1	Mm1
....uaucacagccugcuuugauggU.................................	2	1	Mm1
....uaucacagccugcuuugCuggg.................................	5	1	Mm1
....uaucaGagccugcuuugauggg.................................	1	1	Mm1
....uaucacagccugcuuugGuggg.................................	4	1	Mm1
....uaucacagccugcuGugaugggc................................	1	1	Mm1
....uaucacagccugcuuugGugggc................................	2	1	Mm1
....uaucacagccugcuuugaugggc................................	1493	0	Mm1
....uaucacagccugcuuugaugUgc................................	5	1	Mm1
....uaucacagcUugcuuugaugggc................................	13	1	Mm1
....uaucUcagccugcuuugaugggc................................	1	1	Mm1
....uaucacagccugcuuugaugggG................................	1	1	Mm1
....uaucacagccugcuuugauggUc................................	1	1	Mm1
....uaucacagccugcuCugaugggc................................	2	1	Mm1
....uaucacagccugcCuugaugggc................................	3	1	Mm1
....uaucacagccugcuuuCaugggc................................	4	1	Mm1
....uaucacagccugcuuuUaugggc................................	1	1	Mm1

```
                    Star                                     Mature
augcauacaguuuguauaguccaaugaagugguugugauguguguuggucuauaucauaucacagccugcuuugaugggcuauacugcuguuuucugccaagaaggguucuu
.........................................................uaucacagccugcuuugCugggc................................  10   1  Mm1
.........................................................uaucacagccugcuuugaugggU................................  99   1  Mm1
.........................................................uaucacagccCgcuuugaugggc................................  1    1  Mm1
.........................................................uaucacagccuAcuuugaugggc................................  1    1  Mm1
.........................................................uaucacagAcugcuuugaugggc................................  1    1  Mm1
.........................................................uaucacagccAgcuuugaugggc................................  1    1  Mm1
.........................................................uaucacagUcugcuuugaugggc................................  11   1  Mm1
.........................................................uaucacagccugcAuugaugggc................................  1    1  Mm1
.........................................................uauUacagccugcuuugaugggc................................  1    1  Mm1
.........................................................uaucacagGcugcuuugaugggc................................  1    1  Mm1
.........................................................uaucacGgccugcuuugaugggc................................  1    1  Mm1
.........................................................uaucacagccugcuuugUugggc................................  6    1  Mm1
.........................................................uaucacagccugcuuuAaugggc................................  3    1  Mm1
.........................................................uaucacagccugcuuugaugggA................................  204  1  Mm1
.........................................................uaucacagccugcuuugaugggUu...............................  22   1  Mm1
.........................................................uaucacagccugcuuuAaugggcu...............................  1    1  Mm1
.........................................................uaucacagccugcuuugCugggcu...............................  1    1  Mm1
.........................................................uaucGcagccugcuuugaugggcu...............................  1    1  Mm1
.........................................................uaucacagccugcuuugaugggcG...............................  2    1  Mm1
.........................................................uaucacagccugcuuugUugggcu...............................  1    1  Mm1
.........................................................uaucacagccGgcuuugaugggcu...............................  1    1  Mm1
.........................................................uaucacagccugcuuugaugggcu...............................  359  0  Mm1
.........................................................uaucacagccugcuuugaugggcA...............................  17   1  Mm1
.........................................................uaucacagccugcuuuUaugggcu...............................  1    1  Mm1
.........................................................uaucacagccugcuuugGugggcu...............................  1    1  Mm1
.........................................................uaucacagccAgcuuugaugggcu...............................  1    1  Mm1
.........................................................uaucacagccugcuuugaugggcuU..............................  8    1  Mm1

....................ccaaugUagugguugugaugug......................................................................  1    1  Mm2
....................ccaaugaagugguugugaugug......................................................................  32   0  Mm2
....................ccaaugaagugguugugaugugu.....................................................................  2    0  Mm2
........................ugaagugguugugaugug......................................................................  1    0  Mm2
..........................................uguuggucuauaucauaucacagccugcuu........................................  2    0  Mm2
........................................................Cuaucacagccugcuuugauggg.................................  3    1  Mm2
........................................................auaucacagccugcuuugauggg.................................  8    0  Mm2
........................................................auaucacagccugcuuugaugggc................................  1    0  Mm2
........................................................Cuaucacagccugcuuugaugggc................................  3    1  Mm2
.........................................................uaucacagccugcuuuga.....................................  22   0  Mm2
.........................................................uaucacagccugcuuugU.....................................  2    1  Mm2
.........................................................uaucacagccugcuuugau....................................  35   0  Mm2
.........................................................uaucacagccugcuuugaA....................................  2    1  Mm2
.........................................................uaucacagccugcuuuCau....................................  1    1  Mm2
.........................................................uaucacagccugcuuuUau....................................  1    1  Mm2
.........................................................uaucacagccugcuuuAau....................................  3    1  Mm2
.........................................................uaucacaUccugcuuugau....................................  1    1  Mm2
.........................................................uaucacagccugcuuugUu....................................  6    1  Mm2
.........................................................uaucacagccugcuuuUaug...................................  1    1  Mm2
.........................................................uaucacagccugcuuugauU...................................  1    1  Mm2
.........................................................uaucacagccugcuuugauA...................................  3    1  Mm2
.........................................................uaucacagccugcuuugaug...................................  65   0  Mm2
.........................................................uaucacagccugcuuugCugg..................................  3    1  Mm2
.........................................................uaucacagccugcuuugaugU..................................  2    1  Mm2
.........................................................uaucacagAcugcuuugaugg..................................  1    1  Mm2
.........................................................uaucacagccugcuuugUugg..................................  2    1  Mm2
.........................................................uauAacagccugcuuugaugg..................................  1    1  Mm2
.........................................................uaucacagccGgcuuugaugg..................................  1    1  Mm2
.........................................................uaucacagccugcuuugaCgg..................................  1    1  Mm2
.........................................................uaucacagccugAuuugaugg..................................  1    1  Mm2
.........................................................uaucacagccugcuuugaugg..................................  475  0  Mm2
.........................................................uaucacagccugcuuuUaugg..................................  2    1  Mm2
.........................................................uaucacagccugcuuugGugg..................................  2    1  Mm2
.........................................................uaucacagccuUcuuugaugg..................................  1    1  Mm2
.........................................................uaucacagccAgcuuugaugg..................................  2    1  Mm2
.........................................................uaucacagccugcuuugaugC..................................  2    1  Mm2
.........................................................uaucacagccugcuuugUuggg.................................  28   1  Mm2
.........................................................uaucUcagccugcuuugauggg.................................  2    1  Mm2
.........................................................uaucacagccugcuuugaAggg.................................  2    1  Mm2
.........................................................uaucacagccugUuuugauggg.................................  2    1  Mm2
.........................................................uaucacagccuAcuuugauggg.................................  1    1  Mm2
.........................................................uaucacagccuUcuuugauggg.................................  2    1  Mm2
```

Star Mature

```
augcauacaguuuguauaguccaaugaagugguugugauguguguuggucuauaucauaucacagccugcuuugaugggcuauacugcuguuuucugccaagaaggguucuu
.........................................................uaucacagccugcuuugaCggg.................................   1     1   Mm2
.........................................................uaucacagccCgcuuugauggg.................................   12    1   Mm2
.........................................................uaucacagcUugcuuugauggg.................................   4     1   Mm2
.........................................................uaucCcagccugcuuugauggg.................................   2     1   Mm2
.........................................................uaucacagccugcCuugauggg.................................   2     1   Mm2
.........................................................uaucacagccugcuCugauggg.................................   2     1   Mm2
.........................................................uaAcacagccugcuuugauggg.................................   4     1   Mm2
.........................................................uaucacagccugcuuuUauggg.................................   10    1   Mm2
.........................................................uaucacGgccugcuuugauggg.................................   4     1   Mm2
.........................................................uaucacagccugGuuugauggg.................................   2     1   Mm2
.........................................................uaGcacagccugcuuugauggg.................................   4     1   Mm2
.........................................................uaucGcagccugcuuugauggg.................................   1     1   Mm2
.........................................................uaucacaAccugcuuugauggg.................................   2     1   Mm2
.........................................................uaucaUagccugcuuugauggg.................................   3     1   Mm2
.........................................................uaucacagccGgcuuugauggg.................................   13    1   Mm2
.........................................................Naucacagccugcuuugauggg.................................   3     1   Mm2
.........................................................Caucacagccugcuuugauggg.................................   2     1   Mm2
.........................................................uaucacagccugcuuCgauggg.................................   2     1   Mm2
.........................................................uaucacagUcugcuuugauggg.................................   7     1   Mm2
.........................................................uaucacagccAgcuuugauggg.................................   19    1   Mm2
.........................................................uaucacagccugcuuugCuggg.................................   30    1   Mm2
.........................................................uaucacaCccugcuuugauggg.................................   1     1   Mm2
.........................................................uaucacagccugcuuugauggg.................................   7514  0   Mm2
.........................................................uaucacNgccugcuuugauggg.................................   1     1   Mm2
.........................................................uGucacagccugcuuugauggg.................................   2     1   Mm2
.........................................................uauAacagccugcuuugauggg.................................   2     1   Mm2
.........................................................uaucacagccugcuuugaugUg.................................   1     1   Mm2
.........................................................uaucacagccugcuuugauggA.................................   31    1   Mm2
.........................................................uaucacagccugcuuugauggC.................................   53    1   Mm2
.........................................................uaucacagccugcAuugauggg.................................   2     1   Mm2
.........................................................uaucacaUccugcuuugauggg.................................   3     1   Mm2
.........................................................uaucacagccugcuuugauggU.................................   16    1   Mm2
.........................................................uaucacagccugcuuugauAgg.................................   1     1   Mm2
.........................................................uaucaNagccugcuuugauggg.................................   1     1   Mm2
.........................................................uCucacagccugcuuugauggg.................................   1     1   Mm2
.........................................................uaucacagccugcuuugauUgg.................................   3     1   Mm2
.........................................................uaucacCgccugcuuugauggg.................................   1     1   Mm2
.........................................................uaucacagccugcuuuCauggg.................................   16    1   Mm2
.........................................................uaucacagccugcuAugauggg.................................   1     1   Mm2
.........................................................uaucacagccugcuuuAauggg.................................   20    1   Mm2
.........................................................uaucacagccugcuuugGuggg.................................   20    1   Mm2
.........................................................uaucacagccCgcuuugaugggc................................   10    1   Mm2
.........................................................uUucacagccugcuuugaugggc................................   1     1   Mm2
.........................................................uaucacagccugcuuugaugCgc................................   5     1   Mm2
.........................................................uNucacagccugcuuugaugggc................................   1     1   Mm2
.........................................................uaucacagccugcuuugauggUc................................   5     1   Mm2
.........................................................uaGcacagccugcuuugaugggc................................   3     1   Mm2
.........................................................uaucacagccAgcuuugaugggc................................   12    1   Mm2
.........................................................uaucacagccugcuuuCaugggc................................   17    1   Mm2
.........................................................uaucacagccugcuuuUaugggc................................   10    1   Mm2
.........................................................uaucacaUccugcuuugaugggc................................   9     1   Mm2
.........................................................uaucacagUcugcuuugaugggc................................   15    1   Mm2
.........................................................uaucGcagccugcuuugaugggc................................   2     1   Mm2
.........................................................uaucaUagccugcuuugaugggc................................   2     1   Mm2
.........................................................uaucacagccugcuuuAaugggc................................   18    1   Mm2
.........................................................uaucacagccugcuCugaugggc................................   2     1   Mm2
.........................................................uaucacagccugcuuugaugggG................................   41    1   Mm2
.........................................................uaucacagccugcuuugaugggA................................   898   1   Mm2
.........................................................uaucacagccugcuuugCugggc................................   32    1   Mm2
.........................................................uaucacagccugcuuugauAggc................................   1     1   Mm2
.........................................................uaucacagccugcuuugauUggc................................   3     1   Mm2
.........................................................uaucacGgccugcuuugaugggc................................   9     1   Mm2
.........................................................uauAacagccugcuuugaugggc................................   2     1   Mm2
.........................................................uaAcacagccugcuuugaugggc................................   2     1   Mm2
.........................................................uaucacUgccugcuuugaugggc................................   1     1   Mm2
.........................................................uaucacagccugcuuugaugggU................................   222   1   Mm2
.........................................................uaucacagccugcuuugauggCc................................   1     1   Mm2
.........................................................uaucUcagccugcuuugaugggc................................   1     1   Mm2
.........................................................uaucacagccugcuuugUugggc................................   27    1   Mm2
.........................................................uaucacagccugcuuugaCgggc................................   1     1   Mm2
```

```
                    Star                                 Mature
augcauacaguuuguauaguccaaugaagugguugugauguguguuggucuauaucauaucacagccugcuuugaugggcuauacugcuguuuucugccaagaaggguucuu
.........................................................uaucacagccGgcuuugaugggc................................   7     1   Mm2
.........................................................uaucacagccugcuuugauggAc................................   5     1   Mm2
.........................................................uaucacagccugcuuugGugggc................................   18    1   Mm2
.........................................................uaucacagcUugcuuugaugggc................................   16    1   Mm2
.........................................................uaucacagccugcuuugaugUgc................................   16    1   Mm2
.........................................................uaucacagccugUuuugaugggc................................   2     1   Mm2
.........................................................uaucCcagccugcuuugaugggc................................   2     1   Mm2
.........................................................uGucacagccugcuuugaugggc................................   6     1   Mm2
.........................................................uauUacagccugcuuugaugggc................................   1     1   Mm2
.........................................................uaucacagccugcCuugaugggc................................   7     1   Mm2
.........................................................uaucacagGcugcuuugaugggc................................   1     1   Mm2
.........................................................uaucacagccugcuuugaGgggc................................   1     1   Mm2
.........................................................uaucacagccugcuuugaugggc................................   7899  0   Mm2
.........................................................uaucacagAcugcuuugaugggc................................   1     1   Mm2
.........................................................Caucacagccugcuuugaugggc................................   2     1   Mm2
.........................................................Naucacagccugcuuugaugggc................................   5     1   Mm2
.........................................................uaCcacagccugcuuugaugggc................................   2     1   Mm2
.........................................................uauGacagccugcuuugaugggc................................   1     1   Mm2
.........................................................uaucacagccugcuuCgaugggc................................   1     1   Mm2
.........................................................uaucaNagccugcuuugaugggc................................   1     1   Mm2
.........................................................uaGcacagccugcuuugaugggcu...............................   1     1   Mm2
.........................................................uaucGcagccugcuuugaugggcu...............................   1     1   Mm2
.........................................................uaucaUagccugcuuugaugggcu...............................   2     1   Mm2
.........................................................uaucacagccugcuuugaAgggcu...............................   1     1   Mm2
.........................................................uaucacagccugcuuugaugggAu...............................   30    1   Mm2
.........................................................uaucacagUcugcuuugaugggcu...............................   7     1   Mm2
.........................................................uaucacagccugcuuuAaugggcu...............................   4     1   Mm2
.........................................................uaucacNgccugcuuugaugggcu...............................   1     1   Mm2
.........................................................uaucacagccugcuuugaugggcA...............................   14    1   Mm2
.........................................................uaucacagccugcuuuUaugggcu...............................   4     1   Mm2
.........................................................uaucacagccugcuuugUugggcu...............................   4     1   Mm2
.........................................................uaucacagccugcuuugGugggcu...............................   2     1   Mm2
.........................................................uaucacagccAgcuuugaugggcu...............................   1     1   Mm2
.........................................................uaucacagccCgcuuugaugggcu...............................   3     1   Mm2
.........................................................uaucacagccugcuuuCaugggcu...............................   1     1   Mm2
.........................................................uaucacagccugcuuugaugggcG...............................   60    1   Mm2
.........................................................uaucacagccugcuuugaugggcu...............................   1267  0   Mm2
.........................................................uaucacagccugcuuugCugggcu...............................   5     1   Mm2
.........................................................uaCcacagccugcuuugaugggcu...............................   1     1   Mm2
.........................................................uaucacagcUugcuuugaugggcu...............................   6     1   Mm2
.........................................................uaucacagccugcuuugaugggcC...............................   1     1   Mm2
.........................................................uaucacagccugcuuugaugggUu...............................   21    1   Mm2
.........................................................uaucacagcAugcuuugaugggcu...............................   1     1   Mm2
.........................................................uaucacagccGgcuuugaugggcu...............................   2     1   Mm2
.........................................................uaucacagccugcuuugaugggcuU..............................   18    1   Mm2
.........................................................uaucacagccugcuuugaugAgcua..............................   2     1   Mm2
.........................................................uaucacagccugcuuugaugggcua..............................   4     0   Mm2
.........................................................uaucacagccugcuuugaugggcuUu.............................   13    1   Mm2
.........................................................uaucacagccugcuuugaugggcuau.............................   2     0   Mm2
..........................................................aucacagccugcuuugauggg.................................   3     0   Mm2
..........................................................aucacGgccugcuuugaugggc................................   1     1   Mm2
..........................................................aucacagccugcuuugaugUgc................................   1     1   Mm2
..........................................................aucacagccugcuuugaugggc................................   2     0   Mm2
...........................................................ucacagccugcuuugauggg.................................   1     0   Mm2
...........................................................ucacagccugcuuugaugggc................................   1     0   Mm2
...........................................................ucacagccAgcuuugaugggc................................   1     1   Mm2
............................................................cacagccugcuuugaugggc................................   1     0   Mm2

....................ccaaugaagugguugugUugug......................................................................   2     1   Fm3
....................cAaaugaagugguugugaugug......................................................................   1     1   Fm3
....................ccaaugaagugguugugaugug......................................................................   142   0   Fm3
....................ccaaugaagugguugugCugug......................................................................   1     1   Fm3
....................ccaaugUagugguugugaugug......................................................................   1     1   Fm3
....................ccaaugaagugguugugGugug......................................................................   3     1   Fm3
..........................................uguuggucuauaucauaucacagccu............................................   1     0   Fm3
........................................................auaucacagccugcuuugauggg.................................   14    0   Fm3
.........................................................uaucacagccugcuuuga.....................................   79    0   Fm3
.........................................................uaucacagccugcuuuAa.....................................   1     1   Fm3
.........................................................uaucacagccugcuuuCa.....................................   1     1   Fm3
.........................................................uaucacagccugcuuugU.....................................   13    1   Fm3
```

```
                    Star                                       Mature
augcauacaguuuguauaguccaaugaagugguugugauguguguuggucuauaucauaucacagccugcuuugaugggcuauacugcuguuucugccaagaagguucuu
.........................................................uaucacagccugcuuuUa...................................	1	1	Fm3
.........................................................uaucacagccugcuuugUu..................................	16	1	Fm3
.........................................................uaucacagccugcuuugCu..................................	1	1	Fm3
.........................................................uaucacagccugcuuugGu..................................	1	1	Fm3
.........................................................uaucacagUcugcuuugau..................................	2	1	Fm3
.........................................................uaucacagccugcuuugau..................................	201	0	Fm3
.........................................................uaucacagccugcuuuAau..................................	2	1	Fm3
.........................................................uaucacagccugcuuugaA..................................	3	1	Fm3
.........................................................uaucacagcAugcuuugau..................................	1	1	Fm3
.........................................................uaucacagUcugcuuugaug.................................	5	1	Fm3
.........................................................uaucacagccugcuuugaCg.................................	1	1	Fm3
.........................................................uaucacagccugcuuugauC.................................	1	1	Fm3
.........................................................uaucacagccugcuuugaAg.................................	4	1	Fm3
.........................................................uaucacagccugcuuugauU.................................	5	1	Fm3
.........................................................uaucacagccugcuuugGug.................................	1	1	Fm3
.........................................................uaucacagccugcuuugCug.................................	1	1	Fm3
.........................................................uaucacagccugcuuuUaug.................................	2	1	Fm3
.........................................................uaucacagccGgcuuugaug.................................	1	1	Fm3
.........................................................uaucacagccugcuuugaug.................................	209	0	Fm3
.........................................................uaucacagccugcuuugauA.................................	7	1	Fm3
.........................................................uaucacagcAugcuuugaugg................................	1	1	Fm3
.........................................................uaucacaUccugcuuugaugg................................	4	1	Fm3
.........................................................uaucacagccugcuuugUugg................................	2	1	Fm3
.........................................................uaucacagccugcuuugaugg................................	861	0	Fm3
.........................................................uaucacagccugcuuuCaugg................................	3	1	Fm3
.........................................................uaucacagccAgcuuugaugg................................	2	1	Fm3
.........................................................uaucacagccuUcuuugaugg................................	1	1	Fm3
.........................................................uaucacagccugUuuugaugg................................	2	1	Fm3
.........................................................uaucacagUcugcuuugaugg................................	3	1	Fm3
.........................................................uaucacagccugcuuuAaugg................................	1	1	Fm3
.........................................................uaucacagccugcuuuUaugg................................	2	1	Fm3
.........................................................uaucacagccCgcuuugaugg................................	2	1	Fm3
.........................................................uaucacagccugcuuugCugg................................	5	1	Fm3
.........................................................uaucacagccugcuuugaugU................................	14	1	Fm3
.........................................................uaucacagccGgcuuugaugg................................	4	1	Fm3
.........................................................uaucacagccugcuuugGugg................................	1	1	Fm3
.........................................................uaucacCgccugcuuugauggg...............................	1	1	Fm3
.........................................................uaucacagccugcuuCgauggg...............................	1	1	Fm3
.........................................................uaucacagccugcuuugauggg...............................	12940	0	Fm3
.........................................................uaucacagccugcuuugauggA...............................	34	1	Fm3
.........................................................uauAacagccugcuuugauggg...............................	5	1	Fm3
.........................................................uaucacagccugcuuuCauggg...............................	46	1	Fm3
.........................................................uaucacagUcugcuuugauggg...............................	18	1	Fm3
.........................................................uaucacGgccugcuuugauggg...............................	12	1	Fm3
.........................................................uaucacagccugcuuugUuggg...............................	37	1	Fm3
.........................................................uaucacaUccugcuuugauggg...............................	1	1	Fm3
.........................................................uaucacagccAgcuuugauggg...............................	23	1	Fm3
.........................................................Aaucacagccugcuuugauggg...............................	1	1	Fm3
.........................................................uaucaGagccugcuuugauggg...............................	1	1	Fm3
.........................................................uaucacagcAugcuuugauggg...............................	4	1	Fm3
.........................................................uaucacagccugcuuuUauggg...............................	21	1	Fm3
.........................................................uaucacagccuCcuuugauggg...............................	1	1	Fm3
.........................................................uaucacagccugcAuugauggg...............................	4	1	Fm3
.........................................................uaucacagGcugcuuugauggg...............................	2	1	Fm3
.........................................................uaucacagccugcuuugaugUg...............................	3	1	Fm3
.........................................................uaucaUagccugcuuugauggg...............................	3	1	Fm3
.........................................................uaucacagccugcuuugaCggg...............................	4	1	Fm3
.........................................................uCucacagccugcuuugauggg...............................	1	1	Fm3
.........................................................uauGacagccugcuuugauggg...............................	1	1	Fm3
.........................................................uaucacagccugcuuugaAggg...............................	3	1	Fm3
.........................................................uaAcacagccugcuuugauggg...............................	3	1	Fm3
.........................................................uaucacagccugAuuugauggg...............................	1	1	Fm3
.........................................................uaucacagccugUuuugauggg...............................	9	1	Fm3
.........................................................uaucCcagccugcuuugauggg...............................	1	1	Fm3
.........................................................uaucacagccugcuuuAauggg...............................	29	1	Fm3
.........................................................uaucGcagccugcuuugauggg...............................	4	1	Fm3
.........................................................uaucacagccugcGuugauggg...............................	1	1	Fm3
.........................................................uaucacagccugcuuGgauggg...............................	1	1	Fm3
.........................................................uaucacaCccugcuuugauggg...............................	1	1	Fm3
.........................................................uaucacagccugcuuugCuggg...............................	51	1	Fm3
```

Star **Mature**

augcauacaguuuguauaguccaaugaagugguugugauguguguuggucuauaucauaucacagccugcuuugaugggcuauacugcuguuuucugccaagaaggguucuu			
....uaucacagccugcuuugGuggg.................................	35	1	Fm3
....uaucacagccugcuuugaGggg.................................	1	1	Fm3
....uaucacagccCgcuuugauggg.................................	11	1	Fm3
....uaucacagccGgcuuugauggg.................................	12	1	Fm3
....uaucacagcUugcuuugauggg.................................	8	1	Fm3
....Caucacagccugcuuugauggg.................................	2	1	Fm3
....uGucacagccugcuuugauggg.................................	3	1	Fm3
....uUucacagccugcuuugauggg.................................	1	1	Fm3
....uaucacagccugcCuugauggg.................................	14	1	Fm3
....uaucacagAcugcuuugauggg.................................	1	1	Fm3
....uaucacagccugcuuugauUgg.................................	6	1	Fm3
....uaucacagccugcuuugauggC.................................	3	1	Fm3
....uaucUcagccugcuuugauggg.................................	2	1	Fm3
....uaucacagccugcuuugauggU.................................	39	1	Fm3
....Naucacagccugcuuugauggg.................................	6	1	Fm3
....uaucacagccuAcuuugauggg.................................	1	1	Fm3
....uaucacagccugcuAugauggg.................................	1	1	Fm3
....uaucacagccugcuuAgauggg.................................	1	1	Fm3
....uaucacagcAugcuuugaugggc................................	2	1	Fm3
....uaucGcagccugcuuugaugggc................................	1	1	Fm3
....uaGcacagccugcuuugaugggc................................	8	1	Fm3
....uaucacGgccugcuuugaugggc................................	3	1	Fm3
....uaAcacagccugcuuugaugggc................................	2	1	Fm3
....uaucacagccuCcuuugaugggc................................	1	1	Fm3
....uauAacagccugcuuugaugggc................................	3	1	Fm3
....uaucacagccugcuCugaugggc................................	1	1	Fm3
....Caucacagccugcuuugaugggc................................	1	1	Fm3
....uaucacaUccugcuuugaugggc................................	7	1	Fm3
....uaucacagccugcuuugCugggc................................	47	1	Fm3
....uaucacagccugcGuugaugggc................................	1	1	Fm3
....uaucUcagccugcuuugaugggc................................	1	1	Fm3
....uauGacagccugcuuugaugggc................................	1	1	Fm3
....uaucacagccugcCuugaugggc................................	13	1	Fm3
....uaucacagccugcuuugaugggG................................	17	1	Fm3
....uaucacagGcugcuuugaugggc................................	2	1	Fm3
....uaucacagccugcuuugaugCgc................................	3	1	Fm3
....uaucacagccugcuuCgaugggc................................	1	1	Fm3
....uaucaUagccugcuuugaugggc................................	3	1	Fm3
....uaucacagccugcuuugaugggc................................	10644	0	Fm3
....uaCcacagccugcuuugaugggc................................	9	1	Fm3
....uaucacagccugcuuugGugggc................................	33	1	Fm3
....Naucacagccugcuuugaugggc................................	5	1	Fm3
....uaucacagccugcuuugauggCc................................	3	1	Fm3
....uaucacagccugcAuugaugggc................................	4	1	Fm3
....uaucacagccAgcuuugaugggc................................	14	1	Fm3
....uaucacagccugcuuugauggAc................................	4	1	Fm3
....uaucacNgccugcuuugaugggc................................	1	1	Fm3
....uCucacagccugcuuugaugggc................................	1	1	Fm3
....uaucacagccugUuuugaugggc................................	5	1	Fm3
....uGucacagccugcuuugaugggc................................	2	1	Fm3
....uaucCcagccugcuuugaugggc................................	3	1	Fm3
....uaucacagccuUcuuugaugggc................................	1	1	Fm3
....uaucacagAcugcuuugaugggc................................	1	1	Fm3
....uaucacagccugcuuugUugggc................................	32	1	Fm3
....uaucacagccugcuuugaugggU................................	594	1	Fm3
....uaucacagccugcuuugauggUc................................	2	1	Fm3
....uaucacagccugcuGugaugggc................................	2	1	Fm3
....uaucacagccugcuuugauAggc................................	1	1	Fm3
....uaucacagccugAuuugaugggc................................	1	1	Fm3
....uaucacagUcugcuuugaugggc................................	27	1	Fm3
....uaucacagccugcuuugaugggA................................	2082	1	Fm3
....uaucacagccugcuuuUaugggc................................	16	1	Fm3
....uaucacagccugcuuuCaugggc................................	19	1	Fm3
....uaucacagccuAcuuugaugggc................................	2	1	Fm3
....uauUacagccugcuuugaugggc................................	6	1	Fm3
....uaucacagcUugcuuugaugggc................................	39	1	Fm3
....uaucacagcGugcuuugaugggc................................	1	1	Fm3
....uaucacagccugcuuuAaugggc................................	33	1	Fm3
....uaucacagccGgcuuugaugggc................................	12	1	Fm3
....uaucacagccugcuuugauUggc................................	6	1	Fm3

```
                    Star                                 Mature
augcauacaguuuguauaguccaaugaagugguugugauguguguuggucuauaucauaucacagccugcuuugaugggcuauacugcuguuuucugccaagaaggguucuu
.........................................................uaucacagccCgcuuugaugggc................................   11     1   Fm3
.........................................................uaucacagccugcuuugaugUgc................................   21     1   Fm3
.........................................................uaucacagccugcuuugaCgggc................................   1      1   Fm3
.........................................................uaucacagccugcuuugCugggcu...............................   13     1   Fm3
.........................................................uaucacagccugcAuugaugggcu...............................   1      1   Fm3
.........................................................uaucacagccugcuuugGugggcu...............................   8      1   Fm3
.........................................................uaucUcagccugcuuugaugggcu...............................   1      1   Fm3
.........................................................uaucacagUcugcuuugaugggcu...............................   9      1   Fm3
.........................................................uaucacagccugcuuuUaugggcu...............................   9      1   Fm3
.........................................................uaucacagccugcuuugaugggcu...............................   2667   0   Fm3
.........................................................uaucacagccugcuuugauggCcu...............................   1      1   Fm3
.........................................................uaucacagccugcuuugauggUcu...............................   2      1   Fm3
.........................................................uaucacagccugcuuugaugggUu...............................   68     1   Fm3
.........................................................uaucacagcUugcuuugaugggcu...............................   30     1   Fm3
.........................................................uaucacagccugcCuugaugggcu...............................   1      1   Fm3
.........................................................uauUacagccugcuuugaugggcu...............................   1      1   Fm3
.........................................................uaucaUagccugcuuugaugggcu...............................   2      1   Fm3
.........................................................uaucacagccugcuuugauAggcu...............................   1      1   Fm3
.........................................................uaucacagccugcuuugauUggcu...............................   1      1   Fm3
.........................................................Naucacagccugcuuugaugggcu...............................   1      1   Fm3
.........................................................uaucacagccugcuuuAaugggcu...............................   5      1   Fm3
.........................................................uaucacagccugcuuugaAgggcu...............................   1      1   Fm3
.........................................................uaucacagccugcuuugaugUgcu...............................   1      1   Fm3
.........................................................uaucacagccAgcuuugaugggcu...............................   9      1   Fm3
.........................................................uaucacagccugcuuugaugggcA...............................   9      1   Fm3
.........................................................uaucacagccugcuuuCaugggcu...............................   11     1   Fm3
.........................................................uaucacagccugcuuugaugggAu...............................   63     1   Fm3
.........................................................uaucacagccugcuuugUugggcu...............................   7      1   Fm3
.........................................................uaucacagccCgcuuugaugggcu...............................   3      1   Fm3
.........................................................uaucacagccugcuuugauggAcu...............................   1      1   Fm3
.........................................................uaucacagccGgcuuugaugggcu...............................   3      1   Fm3
.........................................................uaucacGgccugcuuugaugggcua..............................   1      1   Fm3
.........................................................uaucacagccugcuuugaugAgcua..............................   4      1   Fm3
.........................................................uaucacagccugcuuugaugggcuU..............................   9      1   Fm3
.........................................................uaucacagccugcuuugaugggcua..............................   2      0   Fm3
.........................................................uaucacagccugcuuugaugggcuau.............................   1      0   Fm3
..........................................................aucacagccugcuuugauggg.................................   12     0   Fm3
..........................................................aAcacagccugcuuugaugggc................................   1      1   Fm3
..........................................................aucacagccugcuuugaugggc................................   17     0   Fm3
..........................................................aucacagccugcuuugaugggcu...............................   5      0   Fm3
...........................................................ucacagccugcuuugauggg.................................   2      0   Fm3
...........................................................uAacagccugcuuugaugggcu...............................   1      1   Fm3
.............................................................acagccugcuuugaugggc................................   1      0   Fm3
.............................................................acagccugcuuugaugggcu...............................   1      0   Fm3

....................ccaaugaagugguugugaugug......................................................................   6      0   Mm3
................................................ucuauaucauaucacagccugcuuuga.....................................   1      0   Mm3
.......................................................cauaucacagccugcuuugaugggc................................   1      0   Mm3
........................................................auaucacagccugcuuugau....................................   1      0   Mm3
........................................................auaucacagccugcuuugaugg..................................   2      0   Mm3
........................................................auaucacagccugcuuugauggg.................................   7      0   Mm3
........................................................Cuaucacagccugcuuugauggg.................................   1      1   Mm3
........................................................auaucacagccugcuuugaugggA................................   1      1   Mm3
........................................................auaucacagccugcuuugaugggc................................   1      0   Mm3
........................................................Cuaucacagccugcuuugaugggc................................   1      1   Mm3
.........................................................uaucacagccugcuuugU.....................................   2      1   Mm3
.........................................................uaucacagccugcuuuga.....................................   11     0   Mm3
.........................................................uaucacagccugcuuugau....................................   42     0   Mm3
.........................................................uaucacagccugcuuugUu....................................   2      1   Mm3
.........................................................Aaucacagccugcuuugau....................................   1      1   Mm3
.........................................................uaucacagccugcuuugaG....................................   1      1   Mm3
.........................................................uaucacagccugcuuugauU...................................   5      1   Mm3
.........................................................uaucacagccugcuuugaug...................................   55     0   Mm3
.........................................................uaucacagUcugcuuugaug...................................   1      1   Mm3
.........................................................uaucacagccugcuuGgaug...................................   1      1   Mm3
.........................................................uaucacagccugcuuugauA...................................   6      1   Mm3
.........................................................uUucacagccugcuuugaugg..................................   1      1   Mm3
.........................................................uaucacagccugcuuugUugg..................................   1      1   Mm3
.........................................................uaucacagccugcuuugaugg..................................   260    0   Mm3
.........................................................uaucacagccugcuuugaugU..................................   1      1   Mm3
```

```
                          Star                                          Mature
augcauacaguuuguauaguccaaugaagugguugugauguguguuggucuauaucauaucacagccugcuuugaugggcuauacugcuguuuucugccaagaaggguucuu
.........................................................uaucacagccugcuuuCaugg..................................   1      1     Mm3
.........................................................uaucacagccuUcuuugaugg..................................   1      1     Mm3
.........................................................uaucacagccuCcuuugaugg..................................   2      1     Mm3
.........................................................uaucacagccCgcuuugaugg..................................   2      1     Mm3
.........................................................uaucacagccugcuuugGugg..................................   1      1     Mm3
.........................................................uauAacagccugcuuugauggg.................................   1      1     Mm3
.........................................................uaucacagccugcuuugauggU.................................   7      1     Mm3
.........................................................uaucacagccugcuuugauggA.................................   21     1     Mm3
.........................................................uaucacagccugcuuugauggg.................................   2926   0     Mm3
.........................................................uaucacagccugcuCugauggg.................................   1      1     Mm3
.........................................................uaucacGgccugcuuugauggg.................................   6      1     Mm3
.........................................................uaucacagccAgcuuugauggg.................................   8      1     Mm3
.........................................................uaGcacagccugcuuugauggg.................................   1      1     Mm3
.........................................................uaucacagUcugcuuugauggg.................................   2      1     Mm3
.........................................................uaucaNagccugcuuugauggg.................................   1      1     Mm3
.........................................................uaucacagccugcuuugaGggg.................................   1      1     Mm3
.........................................................uaucacUgccugcuuugauggg.................................   2      1     Mm3
.........................................................uaucacagccugcuuugCuggg.................................   9      1     Mm3
.........................................................uaucacagccugcCuugauggg.................................   3      1     Mm3
.........................................................uaucacagccugcuuuAauggg.................................   6      1     Mm3
.........................................................uaucacagccugcuuugauCgg.................................   1      1     Mm3
.........................................................uaucacagccugcuuugauggC.................................   24     1     Mm3
.........................................................uaucacagccCgcuuugauggg.................................   2      1     Mm3
.........................................................uaucacaUccugcuuugauggg.................................   2      1     Mm3
.........................................................uaucacagccugcuuugGuggg.................................   14     1     Mm3
.........................................................uaucacagccugcuGugauggg.................................   2      1     Mm3
.........................................................uaucacagccugcuuugaugUg.................................   1      1     Mm3
.........................................................uaucacaAccugcuuugauggg.................................   1      1     Mm3
.........................................................uaucacagccugcuuugUuggg.................................   8      1     Mm3
.........................................................uaucaUagccugcuuugauggg.................................   2      1     Mm3
.........................................................uaucacNgccugcuuugauggg.................................   1      1     Mm3
.........................................................uaucacagccuUcuuugauggg.................................   1      1     Mm3
.........................................................uaucacagccugcuuuUauggg.................................   4      1     Mm3
.........................................................uaucacagccuCcuuugauggg.................................   1      1     Mm3
.........................................................Naucacagccugcuuugauggg.................................   1      1     Mm3
.........................................................uaucacagcUugcuuugauggg.................................   1      1     Mm3
.........................................................uaucacagccugcAuugauggg.................................   1      1     Mm3
.........................................................uaucacagccugUuuugauggg.................................   2      1     Mm3
.........................................................uaucacagccugcuuCgauggg.................................   1      1     Mm3
.........................................................uaucCcagccugcuuugauggg.................................   1      1     Mm3
.........................................................uaucacagccugcuuuCauggg.................................   5      1     Mm3
.........................................................uaucacagccGgcuuugauggg.................................   3      1     Mm3
.........................................................uaucacagccCgcuuugaugggc................................   3      1     Mm3
.........................................................uaucacagccugcuuugauggUc................................   1      1     Mm3
.........................................................uaucacagccugcuuugaugUgc................................   4      1     Mm3
.........................................................uaucacagccugcuuugaugggU................................   94     1     Mm3
.........................................................uaucacagccugcuuugauggCc................................   8      1     Mm3
.........................................................uaucacagccugcuuugCugggc................................   15     1     Mm3
.........................................................uaucCcagccugcuuugaugggc................................   2      1     Mm3
.........................................................uaucacagccugcCuugaugggc................................   3      1     Mm3
.........................................................uaucacagcUugcuuugaugggc................................   16     1     Mm3
.........................................................uaucacagccugcuuugauggAc................................   1      1     Mm3
.........................................................uaucacagccGgcuuugaugggc................................   1      1     Mm3
.........................................................uaucacagccugcuAugaugggc................................   1      1     Mm3
.........................................................uaucacagccAgcuuugaugggc................................   7      1     Mm3
.........................................................uaucacaUccugcuuugaugggc................................   1      1     Mm3
.........................................................uaGcacagccugcuuugaugggc................................   1      1     Mm3
.........................................................uaucacGgccugcuuugaugggc................................   12     1     Mm3
.........................................................uaucacagccugcuGugaugggc................................   1      1     Mm3
.........................................................Naucacagccugcuuugaugggc................................   1      1     Mm3
.........................................................uaucacagccugcuuugaugggG................................   12     1     Mm3
.........................................................uaucacagccugcuuugaugggc................................   2726   0     Mm3
.........................................................uaucacaAccugcuuugaugggc................................   1      1     Mm3
.........................................................uaucacagccugcuuugGugggc................................   5      1     Mm3
.........................................................uaucacagccugcuuuAaugggc................................   7      1     Mm3
.........................................................uCucacagccugcuuugaugggc................................   1      1     Mm3
.........................................................Caucacagccugcuuugaugggc................................   1      1     Mm3
.........................................................uaucacagccugcuuuUaugggc................................   3      1     Mm3
.........................................................uaucacagccugcuuugUugggc................................   8      1     Mm3
.........................................................uaucacagccugcuuugaugCgc................................   1      1     Mm3
```

Star **Mature**

augcauacaguuuguauaguccaaugaagugguugugauguguguuggucuauaucauaucacagccugcuuugaugggcuauacugcuguuuucugccaagaaggguucuu			
....uaucacagccugcuuugauAggc................................	1	1	Mm3
....uaucacagccugcuuugaugggA................................	427	1	Mm3
....uaucacagccugUuuugaugggc................................	1	1	Mm3
....uaucacagUcugcuuugaugggc................................	10	1	Mm3
....uaucacagccugcuuuCaugggc................................	9	1	Mm3
....uaucacagccugcuuugGugggcu...............................	1	1	Mm3
....uaucacagccugcuuuUaugggcu...............................	1	1	Mm3
....uaucacagccugUuuugaugggcu...............................	1	1	Mm3
....uaucacagccugcuuugaugCgcu...............................	1	1	Mm3
....uaucacagccugcuuugCugggcu...............................	4	1	Mm3
....uaucacagccugcuuugaugggcC...............................	2	1	Mm3
....uaucacagccugcuuuAaugggcu...............................	1	1	Mm3
....uaucacagccugcuuugaugggcA...............................	5	1	Mm3
....uaucacagccugcuuugaugggcG...............................	22	1	Mm3
....uaucacagccugcuuuCaugggcu...............................	3	1	Mm3
....uaucacagccugcAuugaugggcu...............................	1	1	Mm3
....uaucacagccCgcuuugaugggcu...............................	2	1	Mm3
....uaucacagUcugcuuugaugggcu...............................	1	1	Mm3
....uaucacagcUugcuuugaugggcu...............................	5	1	Mm3
....uaucacagccugcuuugaugggUu...............................	4	1	Mm3
....uaucacagcAugcuuugaugggcu...............................	1	1	Mm3
....uaucacagccugcuuugUugggcu...............................	4	1	Mm3
....uaucacGgccugcuuugaugggcu...............................	1	1	Mm3
....uaucacagccugcuuugaugggAu...............................	13	1	Mm3
....uaucacagccugcuuugaugggcu...............................	617	0	Mm3
....uauAacagccugcuuugaugggcu...............................	1	1	Mm3
....uaucacagccugcuuugaugggcuC..............................	1	1	Mm3
....uaucacagccugcuuugaugggcuU..............................	11	1	Mm3
....uaucacagccugcuuugaugggcua..............................	1	0	Mm3
....uaucacagccugcuuugaugggcuUu.............................	4	1	Mm3
....aucacagccugcuuugaugggc................................	4	0	Mm3
....ucacagccugcuuugauggg.................................	1	0	Mm3
....ucacagccugcuuugaugggcu...............................	1	0	Mm3
....cacagccugcuuugaugggc................................	1	0	Mm3
....acagccugcuuugauggg.................................	1	0	Mm3
....acagccugcuuugaugggc................................	1	0	Mm3
....cagccugcuuugaugggc................................	1	0	Mm3
